# A candidate vaccine for human visceral leishmaniasis based on a specific T cell epitope-containing chimeric protein protects mice against *Leishmania infantum* infection

**DOI:** 10.1038/s41541-020-00224-0

**Published:** 2020-08-13

**Authors:** Daniela P. Lage, Patrícia A. F. Ribeiro, Daniel S. Dias, Débora V. C. Mendonça, Fernanda F. Ramos, Lívia M. Carvalho, Daysiane de Oliveira, Bethina T. Steiner, Vívian T. Martins, Luísa Perin, Amanda S. Machado, Thaís T. O. Santos, Grasiele S. V. Tavares, João A. Oliveira-da-Silva, Jamil S. Oliveira, Bruno M. Roatt, Ricardo A. Machado-de-Ávila, Antônio L. Teixeira, Maria V. Humbert, Eduardo A. F. Coelho, Myron Christodoulides

**Affiliations:** 1 Programa de Pós-Graduação em Ciências da Saúde: Infectologia e Medicina Tropical, Faculdade de Medicina, Universidade Federal de Minas Gerais, Belo Horizonte, Minas Gerais Brazil; 2 Laboratório de Imunopatologia, Núcleo de Pesquisas em Ciências Biológicas/NUPEB, Departamento de Ciências Biológicas, Insituto de Ciências Exatas e Biológicas, Universidade Federal de Ouro Preto, Ouro Preto, Minas Gerais Brazil; 3 Programa de Pós-Graduação em Ciências da Saúde, Universidade do Extremo Sul Catarinense, Criciúma, 88806-000 Santa Catarina Brazil; 4 Departamento de Bioquímica e Imunologia, Instituto de Ciências Biológicas, Universidade Federal de Minas Gerais, Belo Horizonte, Minas Gerais Brazil; 5 Neuropsychiatry Program, Department of Psychiatry and Behavioral Sciences, McGovern Medical School, The University of Texas Health Science Center at Houston, Houston, TX USA; 6 Neisseria Research Group, Molecular Microbiology, School of Clinical and Experimental Sciences, University of Southampton Faculty of Medicine, Southampton General Hospital, Southampton, SO16 6YD UK

**Keywords:** Microbiology, Vaccines, Diseases, Infectious diseases, Parasitic infection

## Abstract

Leishmaniases are neglected diseases caused by infection with *Leishmania* parasites and there are currently no prophylactic vaccines. In this study, we designed in silico a synthetic recombinant vaccine against visceral leishmaniasis (VL) called ChimeraT, which contains specific T-cell epitopes from *Leishmania* Prohibitin, Eukaryotic Initiation Factor 5a and the hypothetical LiHyp1 and LiHyp2 proteins. Subcutaneous delivery of ChimeraT plus saponin stimulated a Th1 cell-mediated immune response and protected mice against *L. infantum* infection, significantly reducing the parasite load in distinct organs. ChimeraT/saponin vaccine stimulated significantly higher levels of IFN-γ, IL-12, and GM-CSF cytokines by both murine CD4^+^ and CD8^+^ T cells, with correspondingly low levels of IL-4 and IL-10. Induced antibodies were predominantly IgG2a isotype and homologous antigen-stimulated spleen cells produced significant nitrite as a proxy for nitric oxide. ChimeraT also induced lymphoproliferative responses in peripheral blood mononuclear cells from VL patients after treatment and healthy subjects, as well as higher IFN-γ and lower IL-10 secretion into cell supernatants. Thus, ChimeraT associated with a Th1 adjuvant could be considered as a potential vaccine candidate to protect against human disease.

## Introduction

Leishmaniases are neglected diseases present in 98 countries in the world, with 380 million people exposed to the risk from infection by *Leishmania* parasites (https://www.who.int/leishmaniasis/en/). There are distinct clinical manifestations of disease: tegumentary leishmaniasis, which comprises the cutaneous, mucosal, and cutaneous-diffuse forms, and visceral leishmaniasis (VL), which is a fatal and systemic disease, if left untreated^1^. There are an estimated 700,000 to 1.0 million new human cases of VL reported annually with 50,000 deaths^2^. The parasites can be transmitted to humans through the bite of infected female phlebotomine sandflies^3^ and among the different species known, *Leishmania infantum* is mainly responsible for cases of VL in the Americas, with an estimated incidence of 90% of disease registered in Brazil (https://www.who.int/leishmaniasis/en/). During the last decades, the use of antileishmanial drugs has significantly reduced mortality caused by VL^4,5^. However, challenges remain to be overcome, such as the toxicity, adverse effects and/or high costs of the drugs, as well as the emergence of resistant strains^6,7^. Thus, the research community continues to examine prophylactic vaccination to prevent VL^8,9^.

A cell-mediated immune response against *Leishmania* is believed to protect against infection. It is dependent on the generation of Th1 cells, with the production of pro-inflammatory cytokines, such as interferon-γ (IFN-γ), granulocyte-macrophage colony-stimulating factor (GM-CSF), and interleukin-12 (IL-12), which activate macrophages to kill internalized parasites. Conversely, the generation of a Th2 cell-driven response induces the production of anti-inflammatory cytokines, such as IL-4 and IL-10, which deactivate infected macrophages, thus leading to disease development^10^. There are also other cytokines relevant for the susceptibility or resistance against infection, such as TNF-α, IL-17, among others^9^. In addition, the Th1 or Th2-cell profile is associated with the production of IgG2a and IgG1 isotype antibodies, respectively^11^. Thus, *Leishmania* vaccine candidates should be capable of inducing a Th1 cell-driven response, which should be characterized by IFN-γ, GM-CSF, and IL-12 production, amongst other pro-inflammatory cytokines, as well as by anti-*Leishmania* IgG2a antibodies.

Indeed, in human disease, a depressed cell-mediated immune response has been described that is characterized by the failure of peripheral blood mononuclear cells (PBMC) of the patients to proliferate and produce IFN-γ in response to parasite antigens^12,13^. Antigens able to stimulate a Th1-type response in immune cells of VL patients after treatment are potential candidates for development into VL vaccines^14,15^. To prevent *Leishmania* infection and reduce the number of infected individuals, current research is focused on identifying new prophylactic candidate antigens capable of conferring protection also to non-infected individuals. Such antigens, when used to stimulate cells from healthy individuals not exposed to the parasites, should be considered as prophylactic vaccines candidates^13,16,17^.

Distinct vaccine candidates have been evaluated against VL mainly in murine and/or canine models^18–23^. First-generation vaccines used dead parasites administered with or without adjuvants, whereas second-generation vaccines used genetically-modified parasites or viruses expressing *Leishmania* genes encoding for recombinant proteins. Plasmid DNA-based vaccines that encode genes in eukaryotic expression vectors belong to a third generation^24,25^. However, none of these experimental vaccine candidates have satisfactorily progressed in human trials. Amongst such different vaccine approaches, recombinant protein-based vaccines have several significant advantages, such as stability, low cost, and standardized production^26–28^.

Recently, we showed that immunization with known and hypothetical *Leishmania* proteins produced in recombinant versions protected mice against *L. infantum* infection^22,29,30^. Prohibitin (PHB) is a multifunctional *Leishmania* protein, which is involved in cell proliferation^31^ and maintenance of parasite mitochondrial integrity^32^. The presence of anti-PHB antibodies in *L. donovani*-infected patients showed that it is relevant during active disease and that it could be a potential diagnostic marker for disease^33^. We also showed that recombinant PHB (rPHB) induced a specific Th1 cellular response, characterized by high levels of IFN-γ, IL-12, and GM-CSF in the immunized animals, as well as by proliferative responses specific to the protein, and high IFN-γ levels in PBMC cultures obtained from individuals who had recovered from VL and from healthy subjects^34^. Immunization led to significant reductions in parasite load in the spleen, liver, bone marrow, and draining lymph nodes of the infected and vaccinated animals, compared to unvaccinated control mice.

Eukaryotic Initiation Factor 5a (EIF5a) is a small acidic protein found in prokaryotes and eukaryotes, which is involved in cell proliferation, mRNA decay and retroviral mRNA transport^35^. Notably, EIF5a is antigenic in mammalian hosts, since it was recognized by antibodies in dog sera with VL using an immunoproteomic approach^36^, as well as by antibodies in sera from humans with tegumentary leishmaniasis^37^. We have shown that rEIF5a induced partial protection in immunized mice against *L. infantum* infection, which was related to IFN-γ production mediated by CD4^+^ and CD8^+^ T cells. Similarly, an immunoproteomic study suggested that two other *Leishmania* hypothetical proteins, LiHyp1 and LiHyp2, were also antigenic, since they were recognized by antibodies in VL patients sera, but not by sera from healthy subjects^38^.

Since the repertoire of *Leishmania* proteins is high and variable, production of an experimental vaccine containing multiple immunogens in a unique product seems evident^39^. In the current study, we used immuno-bioinformatics tools to develop a single recombinant chimera protein vaccine (ChimeraT) by combining specific T cell epitopes from PHB, EIF5a, LiHyp1, and LiHyp2 proteins. The protective efficacy induced by ChimeraT in BALB/c mice against *L. infantum* infection, as well as the stimulation of lymphoproliferative responses and cytokine production by PBMC isolated from VL patients before and after treatment and healthy subjects, were evaluated. In addition, the vaccine potential of ChimeraT was compared with the individual recombinant proteins and predicted T-cell epitopes using in vitro and in vivo assays.

## Results

### Constructing and characterizing recombinant ChimeraT

**Fig. 1:**
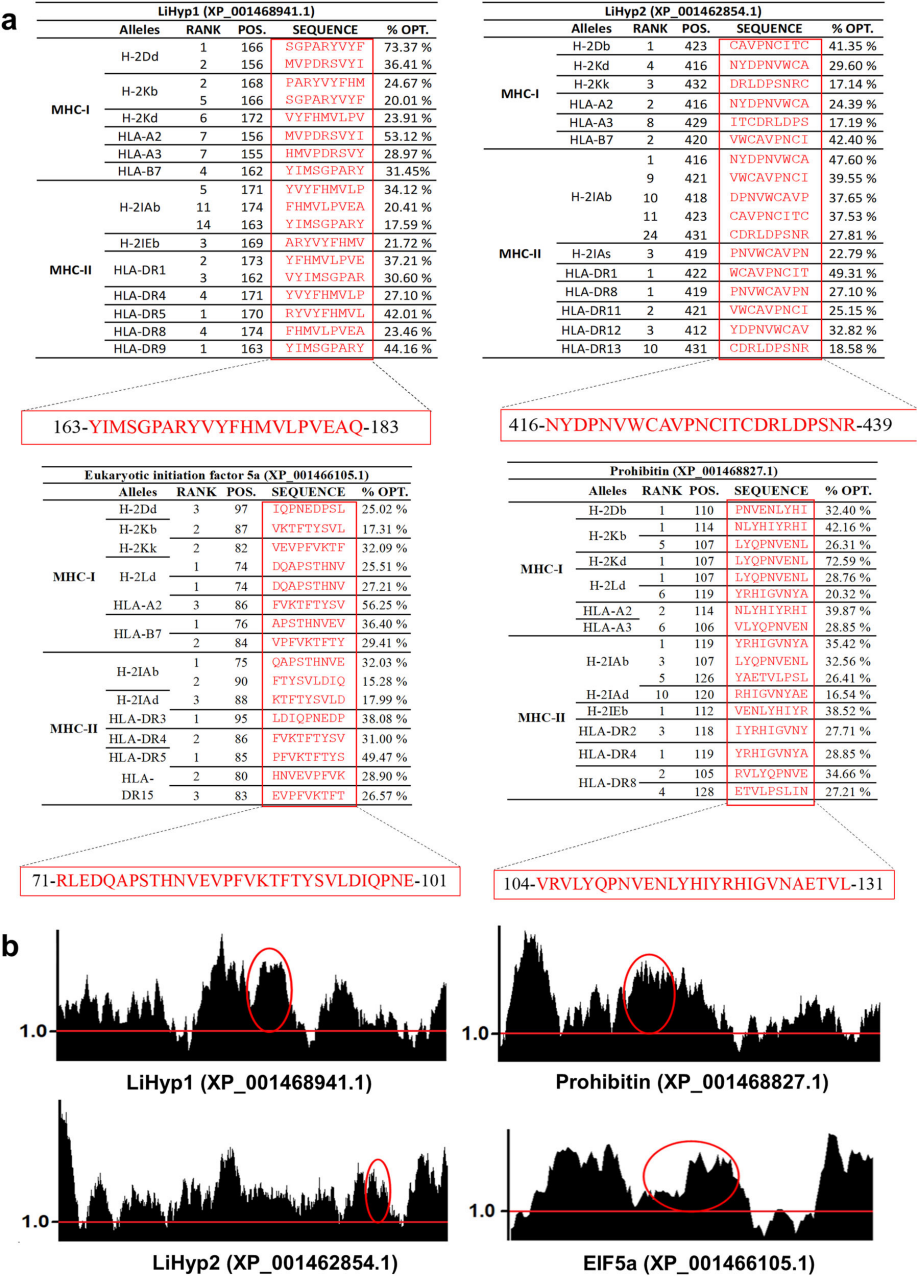
Bioinformatics tools used to construct the ChimeraT protein. **a** Initially, T-cell epitopes (highlighted in red) of Prohibitin (XP_001468827.1), EIF5a (XP_001466105.1), LiHyp1 (XP_001468941.1), and LiHyp2 (XP_001462854.1) proteins were predicted with the Rankpep program and confirmed by NetCTLPan 1.1 and NetMHCII 2.3 programs. **b** Epitope antigenicity was predicted using the online tool integrated into the Immune Epitope Database Analysis Resource (IEDB) database by the Kolaskar & Tongaonkar Antigenicity method. The selected epitopes of Prohibitin (XP_001468827.1), EIF5a (XP_001466105.1), LiHyp1 (XP_001468941.1) and LiHyp2 (XP_001462854.1) proteins are located above the red line and circled in red on the black graphs.

The ChimeraT was constructed using selected CD4^+^ and CD8^+^ T cell epitopes from the amino acid sequences of PHB, EIF5a, LiHyp1, and LiHyp2 proteins. The following epitopes were selected: PHB (amino acids 104–131); EIF5a (amino acids 71–101); LiHyp1 (amino acids 163–183) and LiHyp2 (amino acids 416–439) (Fig. 1a, Table 1). These epitopes were predicted to be antigenic (threshold values >1.0) when evaluated by the IEDB program (Fig. 1b). In the linear amino acid sequence of ChimeraT, two glycine residues were added between the epitopes to enhance solubility of the recombinant protein and the epitopes were arranged to mimic their arrangement in their respective source protein. The physicochemical characteristics of ChimeraT and the individual T-cell epitopes, i.e. number of amino acids, molecular weight, isoelectric point estimate, instability index, aliphatic index, and GRAVY (grand average of hydropathy) values, are shown in Table 1.

**Table 1:** Physiochemical properties of ChimeraT and the individual T-cell epitopes.

Protein/peptide	Amino acid sequence	Number of amino acids	Molecular weight (kDa)	Isoelectric point	Instability index	Aliphatic index	GRAVY
ChimeraT	YIMSGPARYVYFHMVLPVEAQGGRLEDQAPSTHNVEVPFVKTFTYSVLDIQPNEGGNYDPNVWCAVPNCITCDRLDPSNRGGVRVLYQPNVENLYHIYRHIGVNAETVL	109	12.31	5.40	51.03	84.77	−0.245
LiHyp1 epitope	YIMSGPARYVYFHMVLPVEAQ	21	2.47	6.75	50.78	88.10	0.386
EIF5a epitope	RLEDQAPSTHNVEVPFVKTFTYSVLDIQPNE	31	3.54	4.50	57.65	78.39	−0.535
LiHyp2 epitope	NYDPNVWCAVPNCITCDRLDPSNR	24	2.76	4.43	39.18	60.83	−0.667
PHB epitope	VRVLYQPNVENLYHIYRHIGVNAETVL	27	3.21	6.90	54.73	129.63	0.007

*GRAVY* grand average of hydropathy.

ChimeraT was constructed using amino acid sequences containing selected CD4^+^ and CD8^+^ T-cell epitopes from Prohibitin (PHB; Amino Acids 104–131), EIF5a (Amino Acids 71–101), LiHyp1 (Amino Acids 163–183) and LiHyp2 (Amino Acids 416–439) proteins.

### Lymphoproliferation and cytokine production in human cells stimulated with ChimeraT, individual T-cell epitopes, and recombinant proteins

**Fig. 2:**
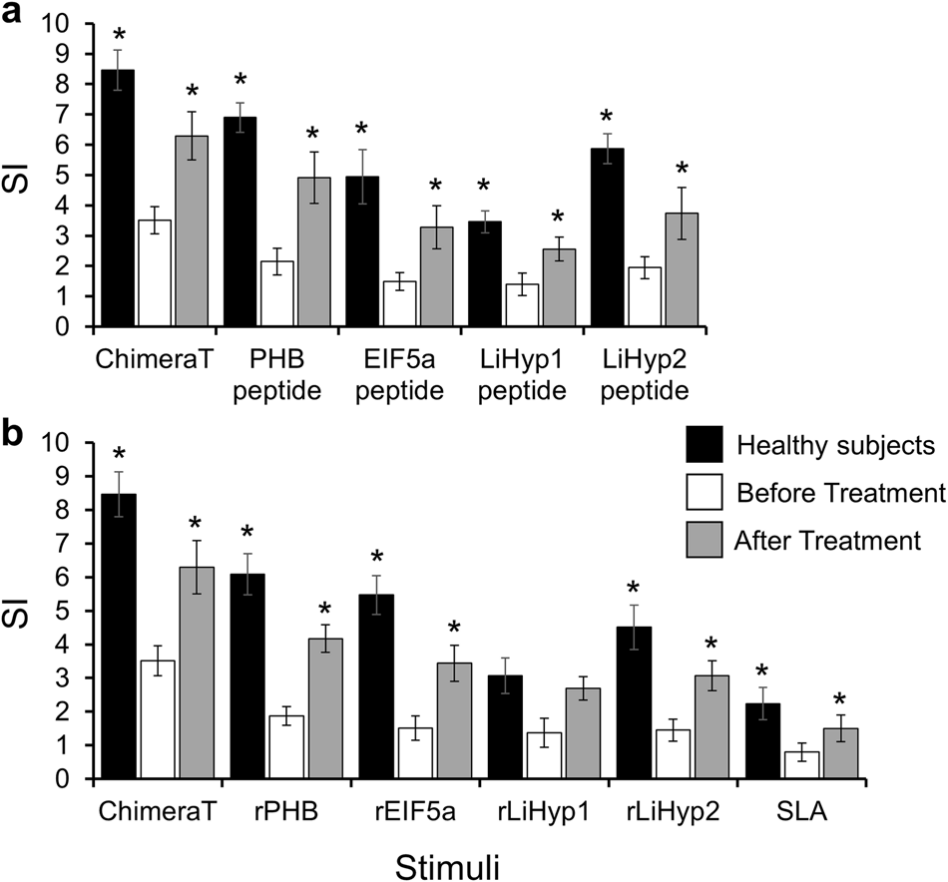
Lymphoproliferation induced by ChimeraT and the specific T-cell epitopes in visceral leishmaniasis patients and healthy subjects. PBMC were isolated from VL patients (*n* = 10) before and after treatment and from healthy subjects (*n* = 10). Induction of lymphoproliferative response (stimulation index, SI) by ChimeraT was compared to the PHB, EIF5a, LiHyp1 or LiHyp2 synthetic peptides (**a**), and the corresponding source recombinant proteins and SLA (**b**). Data are the mean of 10 tested individuals in each group and the error bars denote the standard deviations. (*) indicates statistically significant differences compared to the VL patients before treatment (*P* < 0.001). Statistics were done using one-way analysis of variance (ANOVA), followed by Bonferroni´s post-test, which was used for multiple comparisons, and *P* values < 0.05 were considered significant.

To evaluate the ability of ChimeraT, individual T-cell epitopes, and recombinant proteins to stimulate human cells, PBMCs were purified from blood samples from VL patients before and after treatment and from healthy subjects. The antigens stimulated cellular proliferation from both patients and healthy individuals, as shown by increased stimulation index (SI) values (Fig. 2). Stimulation using ChimeraT induced high lymphoproliferative responses than the individual proteins in VL patients after treatment and healthy subject group (Fig. 2a). This profile was also observed using cells from VL patients before treatment, although values were lower than those observed for the other two groups. In comparison with the recombinant proteins rPHB, rEIF5a, rLiHyp1, and rLiHyp2 and *L. infantum* Soluble *Leishmania* Antigen (SLA), ChimeraT induced significantly higher lymphoproliferation in PBMC isolated from treated patients and healthy subjects (Fig. 2b).

**Fig. 3:**
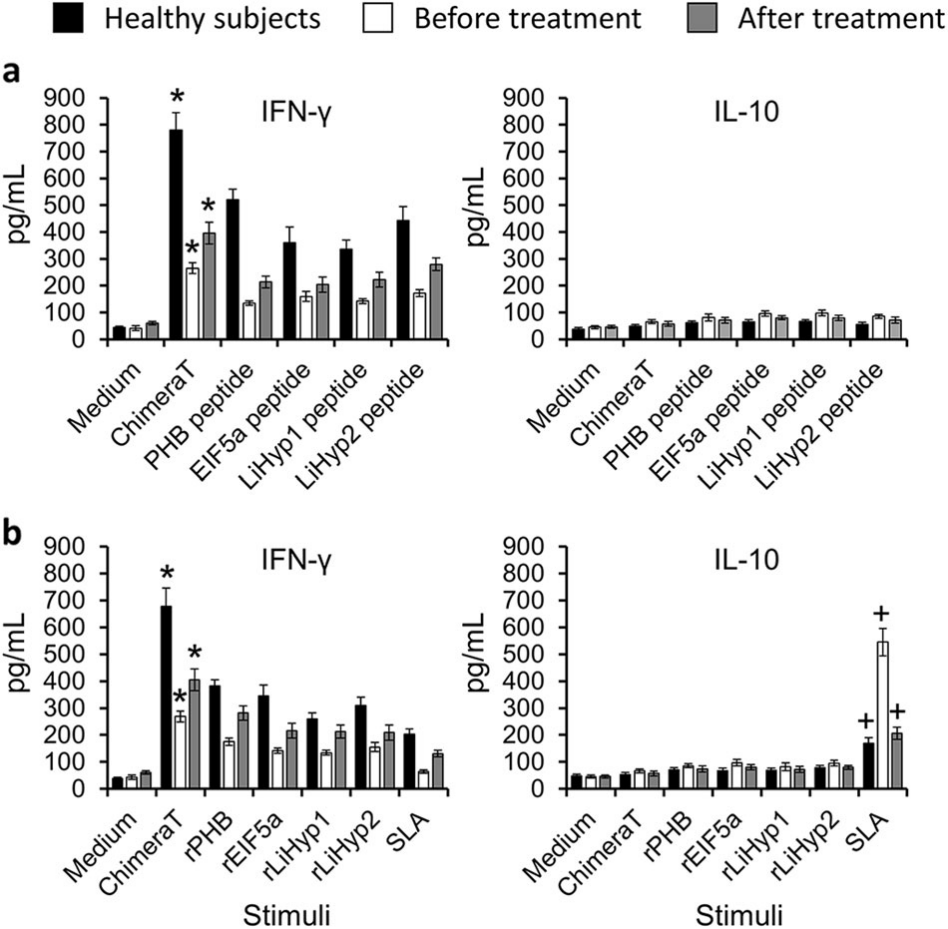
Cytokine production in human PBMC from visceral leishmaniasis patients and healthy subjects. To evaluate specific IFN-γ and IL-10 production in the culture supernatant from VL patients before and after treatment (*n* = 10) and healthy subjects (*n* = 10), PBMC (10^7^ cells per mL) were stimulated with ChimeraT or with PHB, EIF5a, LiHyp1 or LiHyp2 synthetic peptides (**a**) or the corresponding source recombinant proteins and SLA (**b**). Control cells were maintained in medium alone. Cell supernatants were collected and IFN-γ and IL-10 production was measured by capture ELISA. The bars indicate the mean and the error bars denote the standard deviation of the groups. (*) indicates statistically significant differences compared to values obtained from cells stimulated with the individual peptides or recombinant proteins (*P* < 0.05, *P* < 0.01, and *P* < 0.001 respectively; see text). (+) indicates statistically significant differences compared to values obtained from cells stimulated with ChimeraT and individual proteins (*P* < 0.001, *P* < 0.01, and *P* < 0.01 respectively; see text). Statistics were done using one-way analysis of variance (ANOVA), followed by Bonferroni´s post-test, which was used for multiple comparisons, and *P* values < 0.05 were considered significant.

We next examined the ability of ChimeraT, the individual peptides, and recombinant proteins to induce IFN-γ and IL-10 secretion in stimulated PBMC cultures. Compared to stimulation with the individual peptides, ChimeraT induced higher levels of IFN-γ secretion into the cell supernatants of cells from VL patients before and after treatment, and from healthy subjects (*P* < 0.05, *P* < 0.01 and *P* < 0.001, respectively) (Fig. 3a). By contrast, IL-10 levels were low and similar in all groups. Ratios between the IFN-γ and IL-10 levels were calculated, and mean values of ~16.0, ~4.0, and ~7.0 were found for cells from healthy individuals, VL patients before and after treatment, which were stimulated with ChimeraT, respectively. Furthermore, these ratios were significantly higher than those found after stimulation using the individual peptides (*P* < 0.01, *P* < 0.005, and *P* < 0.0001, respectively).

A similar picture was seen when comparing ChimeraT and the individual recombinant proteins and SLA (Fig. 3b). Stimulation with the ChimeraT induced significantly higher IFN-γ levels into the cell supernatants of cells from VL patients before and after treatment, and from healthy subjects (*P* < 0.05, *P* < 0.01, and *P* < 0.005, respectively), as compared to the values using the individual proteins as stimuli (Fig. 3b). By contrast, SLA induced the production of significantly higher IL-10 levels in stimulated cell cultures from VL patients before and after treatment, and from healthy subjects, as compared to the use of ChimeraT and individual proteins (*P* < 0.001, *P* < 0.01, and *P* < 0.01, respectively). Ratios between IFN-γ and IL-10 levels were also calculated, and cells from healthy individuals, VL patients before and after treatment, stimulated with ChimeraT showed significantly higher ratios (~12, ~4, and 7 respectively) than those observed after stimulation with individual proteins and SLA (*P* < 0.05, *P* < 0.01, and *P* < 0.001, respectively).

### Immunization with ChimeraT prevents the development of infection in mice

**Fig. 4:**
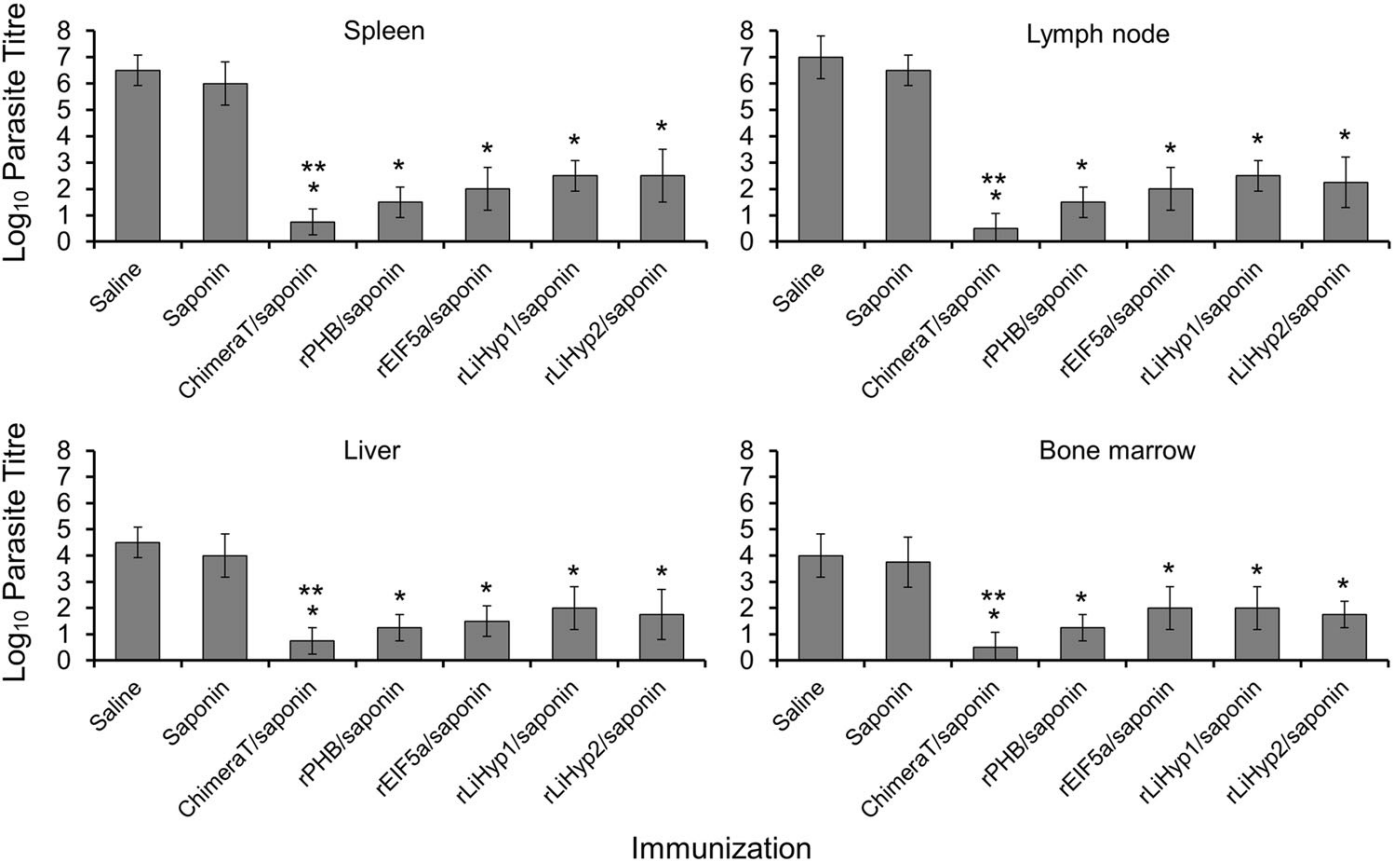
Evaluation of the parasite burden. BALB/c mice (*n* = 8 per group) were immunized with ChimeraT, rPHB, rEIF5a, rLiHyp1 or rLiHyp2 (15 μg each per mouse) mixed with saponin. Controls received saline or saponin alone. Thirty days after the last vaccine dose, mice were challenged with 10^7^*L. infantum* stationary promastigotes and, 45 days after infection, their spleens, livers, draining lymph nodes, and bone marrows were collected and the parasite load was quantified by a limiting-dilution technique. Data were expressed as the negative log of the titer adjusted per milligram of organ; the columns indicate the means and the error bars, the standard deviations. (*) indicates statistically significant difference compared to the saline and saponin groups (*P* < 0.0001). (**) indicates statistically significant difference compared to the rEIF5a/saponin, rLiHyp1/saponin and rLiHyp2/saponin groups (*P* < 0.05). Statistics were done using one-way analysis of variance (ANOVA), followed by Bonferroni´s post-test, which was used for multiple comparisons, and *P* values < 0.05 were considered significant.

BALB/c mice were immunized with ChimeraT or the corresponding whole recombinant proteins (rPHB, rEIF5a, rLiHyp1, and rLiHyp2) delivered with saponin adjuvant and 30 days after the last vaccine dose, they were challenged with *L. infantum* promastigotes. The spleens, livers, bone marrows, and draining lymph nodes were collected at 45 days after infection and parasitism was evaluated by a limiting-dilution technique. Compared to the control groups (saline and saponin), immunization with rPHB/saponin, rEIF5a/saponin, rLiHyp1/saponin, and rLiHyp2/saponin significantly reduced parasite loads in the spleen, liver, bone marrow and draining lymph nodes, by log orders of magnitude of ~2.5–4.0, ~2.0–3.0, ~2.0–3.0, and ~3.0–4.5, respectively (*P* < 0.0001) (Fig. 4). Notably, immunization with ChimeraT/saponin led to significantly greater reductions in parasite burden by ~6.0–9.0 log orders of magnitude, when compared to the control groups (*P* < 0.0001). Furthermore, immunization with ChimeraT/saponin induced significant reduction in parasitism when compared to immunization with the rEIF5a/saponin, rLiHyp1/saponin, and rLiHyp2/saponin groups (*P* < 0.05), but was not statistically different to the rPHB/saponin group (*P* ≥ 0.05) (Fig. 4).

### Cellular response generated before and after *L*. *infantum* infection

**Fig. 5:**
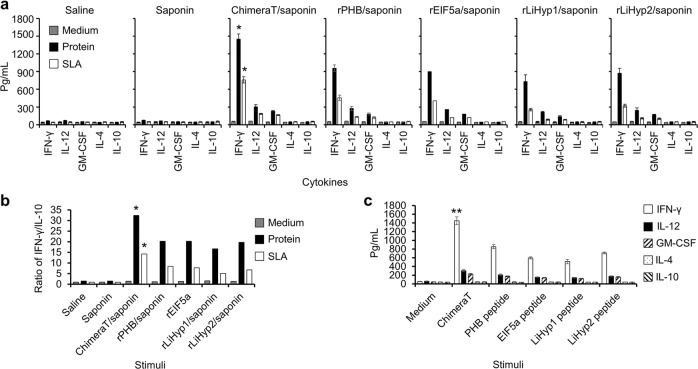
Cytokine responses before *L. infantum* infection. BALB/c mice (*n* = 8 per group) were immunized with ChimeraT, rPHB, rEIF5a, rLiHyp1 or rLiHyp2 (15 μg each per mouse) mixed with saponin. Controls received saline or saponin alone. Thirty days after the last vaccine dose, their spleen cells were collected and cultured (5 × 10^6^ cells per mL) in DMEM (Medium) or stimulated with each homologous protein used for immunization (10 µg/mL each) or *L. infantum* SLA (50 µg/mL) for 48 h at 37 °C with 5% (v/v) CO_2_. **a** Measurement of IFN-γ, IL-12, GM-CSF, IL-4, and IL-10 cytokine levels in cell supernatants. **b** Ratios between mean IFN-γ and IL-10 levels. **c** Evaluation of IFN-γ, IL-12, GM-CSF, IL-4, and IL-10 production in spleen cells of the Chimera T/saponin-immunized mice stimulated in vitro with synthetic peptide epitopes. The bars indicate the mean and the error bars denote the standard deviation of the groups. (*) indicates significant difference in relation to the groups of mice immunized with the recombinant proteins plus adjuvant (*P* < 0.005). (**) indicates significant difference in relation to using the individual peptides as stimuli (*P* < 0.01). Statistics were done using one-way analysis of variance (ANOVA), followed by Bonferroni´s post-test, which was used for multiple comparisons, and *P* values < 0.05 were considered significant.

**Fig. 6:**
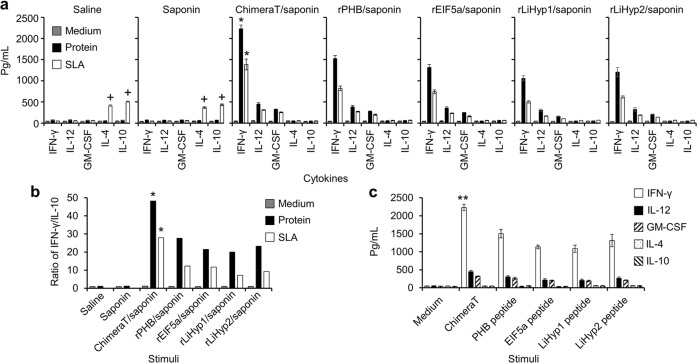
Cytokine production after *L. infantum* challenge. BALB/c mice (*n* = 8 per group) immunized with ChimeraT, rPHB, rEIF5a, rLiHyp1 or rLiHyp2 (15 μg each per mouse) plus saponin were challenged with 10^7^*L. infantum* stationary promastigotes and, 45 days after infection, their spleens were collected. Spleen cells (5 × 10^6^ per mL) were cultured in DMEM (medium, control) or stimulated with each immunizing protein (10 µg/mL each) or *L. infantum* SLA (50 µg/mL) for 48 h at 37 °C with 5% (v/v) CO_2_. **a** Measurement of IFN-γ, IL-12, GM-CSF, IL-4, and IL-10 cytokine levels in cell supernatants. **b** Ratios between the IFN-γ and IL-10 levels. **c** Evaluation of IFN-γ, IL-12, GM-CSF, IL-4, and IL-10 production in spleen cells from the Chimera T/saponin-immunized mice stimulated in vitro with the synthetic peptide epitopes. The bars indicate the mean and the error bars denote the standard deviation of the groups. (*) indicates significant difference in relation to the groups of mice immunized with the recombinant proteins plus adjuvant and challenged with *L. infantum* parasites (*P* < 0.005). (**) indicates significant difference in relation to using the individual peptides as stimuli (*P* < 0.01). (+) indicates significant difference in relation to the groups of mice immunized with the Chimera T or recombinant proteins plus adjuvant and challenged with *L. infantum* parasites (*P* < 0.001). Statistics were done using one-way analysis of variance (ANOVA), followed by Bonferroni´s post-test, which was used for multiple comparisons, and *P* values < 0.05 were considered significant.

The cellular response developed after immunization with the recombinant proteins and ChimeraT was evaluated in BALB/c mice, before and after *L. infantum* challenge. For this evaluation, spleen cells were collected from mice 30 days after the last vaccine dose and before challenge (Fig. 5), as well as 45 days after infection (Fig. 6). Cells were stimulated in vitro with the homologous antigen, i.e. ChimeraT or the recombinant proteins, as well as with the synthetic peptides and *L. infantum* SLA. Before challenge, stimulated spleen cells of the ChimeraT and recombinant protein-immunized mice produced significantly higher levels of IFN-γ, IL-12 and GM-CSF, compared with the values obtained in the control (saline and saponin) groups (Fig. 5a). Moreover, immunization with ChimeraT followed by homologous stimulation or with SLA stimulation induced higher IFN-γ levels, when compared to the values obtained in the groups immunized with the recombinant proteins plus adjuvant. Ratios between IFN-γ and IL-10 levels also showed the higher immunogenicity of ChimeraT (ratio ~35.0) over the recombinant proteins (ratios ~15.0–20.0) (Fig. 5b). In addition, stimulation of spleen cells with Chimera T induced significantly higher IFN-γ than that induced using the individual peptides as stimuli (Fig. 5c). In all cases, IL-4 and IL-10 production was low and similar in all groups.

After challenge, the Th1 cellular response profile was maintained in mice immunized with ChimeraT and the recombinant proteins, with significantly increased levels of IFN-γ, IL-12 and GM-CSF, associated with low IL-4 and IL-10 production, as compared to the saline and saponin groups (Fig. 6a). Moreover, ChimeraT/saponin-immunized mice showed significantly higher levels of IFN-γ, when their spleen cells were stimulated with the ChimeraT or SLA, as compared to data obtained in the groups immunized with the recombinant proteins plus adjuvant (Fig. 6a). Conversely, splenocytes collected from mice injected with saline or saponin produced significantly higher levels of IL-4 and IL-10 when SLA was used as a stimulus, in comparison to the other groups. The increased ratio between IFN-γ and IL-10 cytokines again demonstrated the higher immunogenicity of ChimeraT (ratio ~50.0) over the recombinant proteins (ratios ~20.0–25.0) (Fig. 6b). In addition, the Th1-type response was maintained with in vitro stimulation of spleen cells with ChimeraT and the peptide epitopes, with cytokine levels significantly increasing after challenge, when ChimeraT was used as stimulus, in comparison to the values obtained with the individual peptides (*P* < 0.05) (Fig. 6c). Taken together, data for splenic cytokine production obtained before and after parasite challenge demonstrated that immunization with ChimeraT/saponin induced a more polarized Th1-type response profile than immunization with rPHB/saponin, rEIF5a/saponin, rLiHyp1/saponin or rLiHyp2/saponin.

**Fig. 7:**
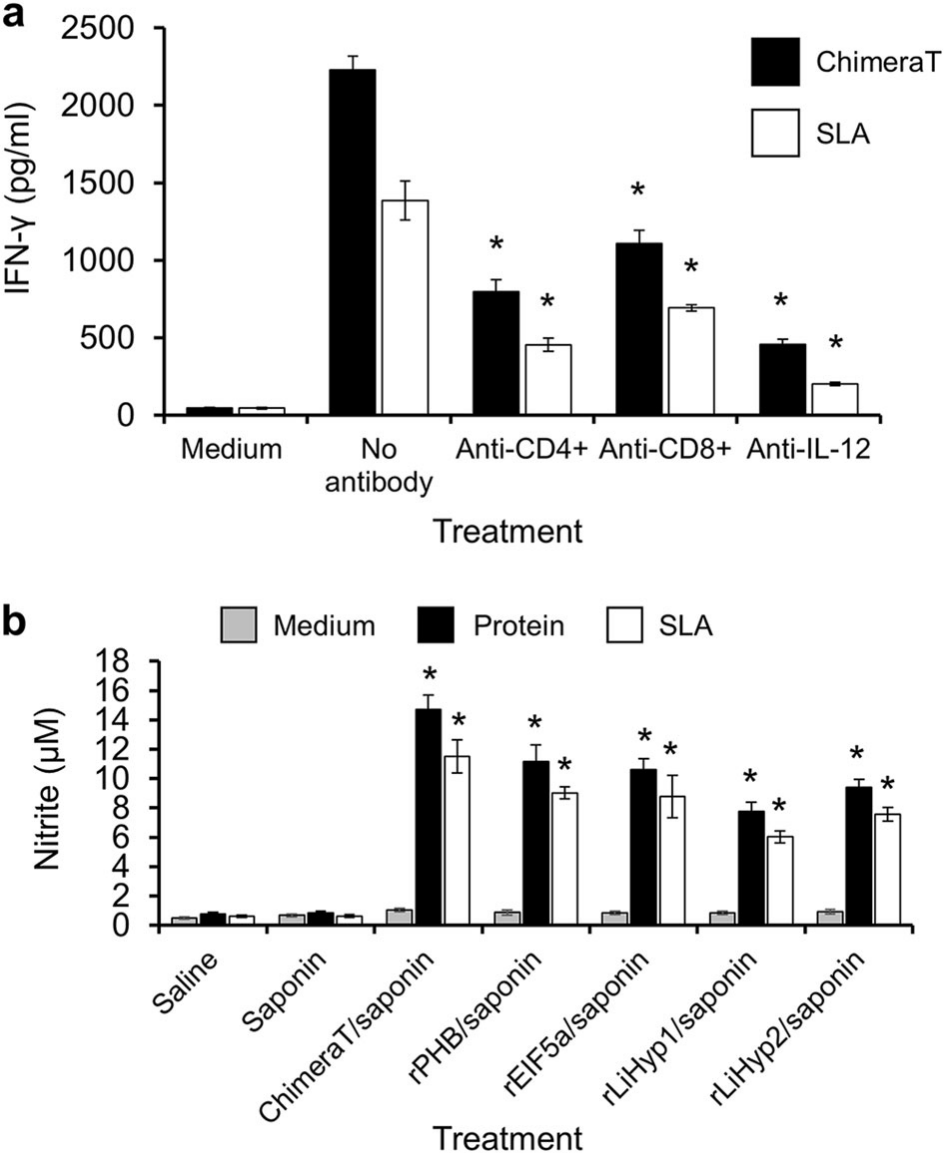
Involvement of CD4^+^ and CD8^+^ T-cell subtypes in the IFN-γ and nitrite secretion. **a** Quantification of IFN-γ production levels in T cell supernatants of the ChimeraT/saponin-immunized mice and challenged with *L. infantum*. Cells were stimulated with ChimeraT or SLA (10 and 50 µg/mL, respectively) in the absence (medium, control) or presence of anti-CD4^+^, anti-CD8^+^ or anti-IL-12 monoclonal antibodies for 48 h at 37 °C with 5% (v/v) CO_2_. **b** Nitrite secretion levels in the culture supernatants of all infected and vaccinated mice, evaluated in vitro by the Griess method. The bars indicate the mean and the error bars denote the standard deviation of the groups. In **a** (*) indicates statistically significant difference compared with no antibody treatment (*P* < 0.0001). In **b** (*) indicates statistically significant difference compared with the control saline and saponin groups (*P* < 0.0001). Statistics were done using one-way analysis of variance (ANOVA), followed by Bonferroni´s post-test, which was used for multiple comparisons, and *P* values < 0.05 were considered significant.

The participation of CD4^+^ and CD8^+^ T cell subtypes in producing IFN-γ in the ChimeraT/saponin group was evaluated by adding monoclonal antibodies to block production of this cytokine in the stimulated cultures. Results showed significantly lower values of IFN-γ, when CD4^+^ and CD8^+^ cells were stimulated with ChimeraT and blocked with the specific monoclonal antibodies, in the order of 65 and 50%, respectively (Fig. 7a). In addition, IFN-γ levels produced by T-cell subtypes stimulated with the SLA were reduced by 67 and 50%, respectively (Fig. 7a). Nitrite (NO_2_^-^) production was evaluated by Griess reaction, and homologous stimulation of spleen cells from mice immunized with ChimeraT/saponin and the recombinant proteins plus saponin induced significantly higher levels of this molecule, when compared to the values obtained in the control groups. Higher NO_2_^−^ levels were observed also in the cell supernatants of the ChimeraT/saponin-immunized mice compared to the individual proteins, peptides or SLA (Fig. 7b).

### Presence of IgG1 and IgG2a isotype antibody in immunized mice, before and after *L. infantum* infection

**Fig. 8:**
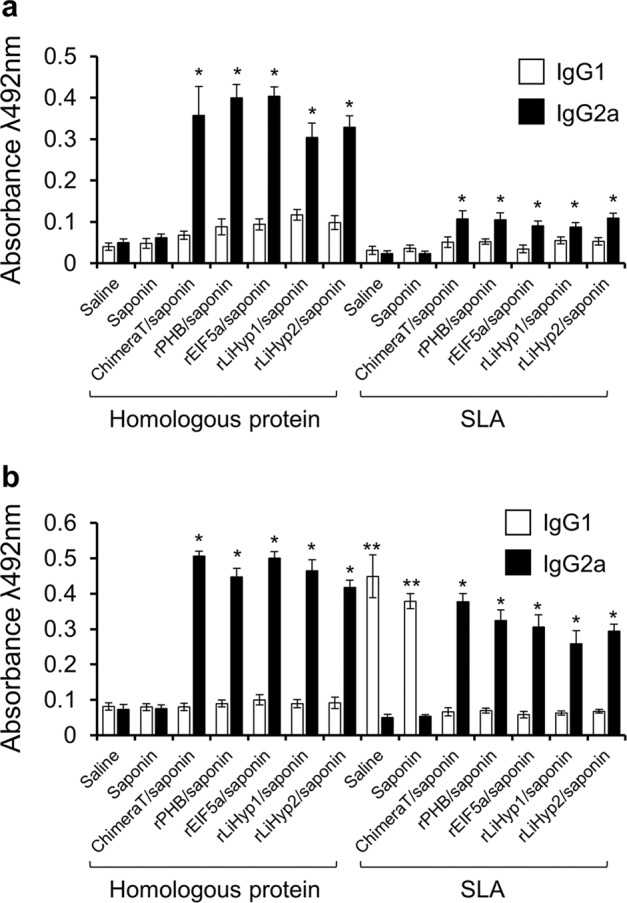
IgG1 and IgG2a antibody isotype levels in immunized mice before and after *L. infantum* infection. BALB/c mice (*n* = 16 per group) were immunized with ChimeraT, rPHB, rEIF5a, rLiHyp1 or rLiHyp2 mixed with saponin adjuvant and, 30 days after the last vaccine dose, serum samples were collected from 8 animals per group. The remaining mice (*n* = 8 per group) were challenged and, 45 days after infection, their serum samples were also collected. IgG1 and IgG2a isotype antibody levels were measured against the homologous proteins and SLA by an ELISA, **a** before infection and **b** after infection. The bars indicate the mean and the error bars denote the standard deviation of the groups. In **a** (*) indicates significant difference in relation to the control saline and saponin control groups (*P* < 0.0001). In **b** (**) indicates statistically significant difference in relation to the recombinant proteins/saponin groups (*P* < 0.0001). Statistics were done using one-way analysis of variance (ANOVA), followed by Bonferroni´s post-test, which was used for multiple comparisons, and *P* values < 0.05 were considered significant.

The humoral response was evaluated in sera from immunized animals before and after challenge with *L. infantum*. Before challenge, immunization with ChimeraT/saponin and the recombinant proteins plus saponin all induced significantly higher homologous protein-specific IgG2a levels than the control groups (Fig. 8a). The IgG2a levels were also significantly higher than IgG1 levels induced by the same antigens. Although SLA-specific antibody production was lower in all of the groups, those immunized with ChimeraT and the recombinant proteins still showed predominance of the IgG2a isotype (Fig. 8a). After infection, the higher levels of IgG2a and lower levels of IgG1 were maintained against ChimeraT and the homologous proteins, but increased levels of parasite-specific IgG2a antibodies were now observed (Fig. 8b). As expected, the control mice challenged with *L. infantum* produced significantly higher levels of SLA-specific anti-parasite IgG1 antibodies, when compared to the IgG2a levels (Fig. 8b).

## Discussion

In the present study, we designed in silico a recombinant multi-epitope vaccine for VL named ChimeraT. The vaccine contains amino acid sequences corresponding to specific T-cell epitopes from four *L. infantum* proteins, which we previously showed to be antigenic in canine and/or human disease^27,34,38^. The most significant findings were that ChimeraT (i) protected mice against *L. infantum* infection and (ii) induced a Th1-type immune response in PBMC from VL patients after treatment and healthy subjects, which was characterized by high IFN-γ and low IL-10 levels and positive lymphoproliferative responses. The protective efficacy of ChimeraT validates the use of specific T-cell epitopes grouped into a single recombinant chimeric molecule as potential vaccine candidates, and is based on recent advances in understanding the mechanisms of cellular immunity in leishmaniasis^40^. Multiple epitope-based vaccines present potential advantages when compared with vaccines using single or combinations of recombinant proteins. We showed that vaccine design can be tailored by selecting the most antigenic CD4^+^ and CD8^+^ T-cell epitopes from immunogenic *Leishmania* proteins and thus avoiding epitopes present in whole proteins with low immunogenicity or undesirable immunodominance^41^. Other advantages of multiple epitope-based vaccines include safety, reduced cost of manufacture, facile quality control, and the opportunity to rationally engineer the epitopes to further increase their immunological potency^39,42^.

In our current study, the rationale for selecting T-cell epitopes from *Leishmania* PHB, EIF5a, LiHyp1, and LiHyp2 proteins is based on our previous studies that demonstrated their vaccine and/or diagnostic potential^27,34,38^. We confirmed the protective activities of the individual recombinant proteins, but demonstrated clearly that ChimeraT was superior. Lymphoproliferation and cytokine production were evaluated as a measure of T-cell activation, and our data showed that stimulation with ChimeraT induced higher lymphoproliferation and IFN-γ production than the individual recombinant proteins or synthetic peptide epitopes. Moreover, higher responses were obtained with PBMC from healthy individuals and from VL patients after treatment than the responses observed from VL patients before treatment: this observation suggests the use of ChimeraT as a possible immunogen to protect against VL in humans. Our findings are in agreement with previous reports showing that PBMC of VL patients after treatment that were stimulated in vitro with other *Leishmania* protein, e.g. rTriose Phosphate Isomerase, rProtein Disulfide Isomerase or rElongation Factor-2, all in combination with rHSP70^43^, or recombinant Elongation Factor-2, Enolase, Aldolase, Triose Phosphate Isomerase, Protein Disulfide Isomerase or p45 proteins^44^, induced high IFN-γ and IL-12 levels, as well as low IL-10 production, when compared to disease active patients. Furthermore, Joshi et al.^41^ recently reported that three individual T-cell epitopes (derived from Enolase, P-14 and Triose Phosphate Isomerase, but not linked together in a chimera) induced significant lymphoproliferative and Th1-biased cytokine responses both in golden hamsters and healthy subjects. Immunization of golden hamsters with such peptides plus Bacillus Calmette-Guerin (BCG) as adjuvant (tuberculosis vaccine) showed ~75% protective efficacy against *L. donovani* infection. Our current study also demonstrates the vaccine potential of a T-cell epitope-based *Leishmania* chimeric antigen and its improved protective efficacy by combining multiple epitopes into a single molecule, which facilitates easier manufacture, quality control, and delivery. Moreover, the protective responses induced by ChimeraT were achieved using a single moiety adjuvant saponin, and not with a complex multi-component adjuvant mixture, such as BCG.

In our study, we also examined the cytokine production profile amongst vaccinated mice, before and after *L. infantum* challenge. The secretion of pro-inflammatory IFN-γ, IL-12, and GM-CSF cytokines was significantly higher in ChimeraT-immunized mice when compared to immunization with the individual recombinant proteins delivered with the same adjuvant. In general, although the levels of murine anti-inflammatory IL-4 and IL-10 cytokines were low in all groups before infection, examination of the IFN-γ/IL-10 ratios showed that the values were higher following ChimeraT/saponin immunization when compared to the individual proteins. These data clearly demonstrate an enhanced Th1-type immune activation induced by ChimeraT/saponin, which is of prime importance since IFN-γ production is crucial for host clearance of intracellular pathogens, such as *Leishmania*^40^. By contrast, IL-10 is considered a Th2-type cytokine and its production is contra-indicative for protection against *Leishmania*^13,45^.

The involvement of T cell subtypes in IFN-γ production was evaluated in mice vaccinated with ChimeraT/saponin. Our data showed that CD4^+^ and CD8^+^ T cell subtypes appeared to have an important role in the production of this cytokine. A limitation of our study is that our experiments were based on inhibition of IFN-γ production by treatment with specific antibodies against CD4^+^ and CD8^+^ molecules, although this experimental strategy has been used in several reports on the immunogenicity of other *Leishmania* antigens^34,46–48^. Additional support for our findings could be obtained with intracellular cytokine staining and quantification with flow cytometry to determine the proportion of both IFN-γ-producing T cell subtypes and their contribution to vaccine efficacy.

Another key finding from our study was that immunization with ChimeraT and the individual recombinant proteins induced predominantly anti-*Leishmania* IgG2a antibodies, which was also associated with a Th1-type immune response. Although ChimeraT was constructed with predicted T cell epitopes of four immunogenic *Leishmania* proteins, our study showed that ChimeraT/saponin induced not only a cellular immune response, but also a humoral immune responses in vaccinated mice. This observation agrees with other studies that reported the development of a humoral immune response specific to recombinant chimerae used to immunize mice^39,49^. It is probable that this humoral response profile, based on anti-ChimeraT and anti-parasite IgG2a isotype antibody, is correlated with IFN-γ production and with the protective phenotype observed against *L. infantum* infection.

We also measured NO_2_^−^ production as a proxy for nitric oxide (NO) and showed that spleen cells from mice immunized with ChimeraT and the recombinant proteins produced significant NO_2_^−^ after stimulation with homologous antigen. This is another important attribute for the ChimeraT vaccine, since the production of this molecule by the inducible NO synthase is believed to be a key defense mechanism against *Leishmania*^50^. In addition, secretion of GM-CSF as an activation marker of macrophages was also increased in the protected and vaccinated animals. However, TNFα, which is a cardinal cytokine for macrophage activation, as well as a broader range of other inflammatory cytokines, could also have been measured and reflects a limitation of our study. Nevertheless, GM-CSF is an important cytokine associated with macrophage activation, resistance and maturation, against different intracellular pathogens including *L. major*^51^, *L. donovani*^52^, and *L. infantum*^22,27,46^. Our current study also agrees with a previous study that showed that BALB/c mice vaccinated with *Leishmania* ribosomal proteins (LRP) plus saponin produced high levels of GM-CSF when compared to the control groups. This high production of GM-CSF was also maintained after infection in the vaccinated animals and was LRP- and SLA-specific^53^. In addition, immunization of human volunteers with a crude *Leishmania* preparation using GM-CSF as an adjuvant induced a parasite-specific Th1 response^54^ and administration of a therapeutic vaccine containing four *Leishmania* antigens combined with GM-CSF correlated with the resolution of mucosal lesions present in an antimonial-refractory mucosal leishmaniasis patient^55^.

ChimeraT represents our latest attempt to develop a chimera vaccine using an in silico approach. Our first Recombinant Chimeric Protein vaccine (RCP1, 268 amino acids with a relative molecular mass (*Mr*) of 31.6 kDa) was constructed with specific CD4+ and CD8+ T-cell epitopes from the hypothetical amastigote-specific proteins LiHyp1 and LiHyp6, from the conserved hypothetical protein LiHyV and from the IgE-dependent histamine-releasing factor HRF^49^. Our second Recombinant Chimera Protein vaccine (RCP2, 422 amino acids with a *Mr* of 47.0 kDa) was constructed with T-cell epitopes from PHB, Small Glutamine-rich Tetratricopeptide repeat-containing protein (SGT) and *Leishmania* hypothetical protein LiHyS^39^. However, the amino acid sequences chosen from all the source proteins to produce RCP1 and RCP2 were larger polypeptides, including for the two antigens that are shared with ChimeraT. Similar to ChimeraT, immunization with RCP1 and RCP2 also developed Th1-type immune responses, which were characterized by high IFN-γ derived from both T-cell subtypes, low IL-10 levels, and significant reductions in the parasite burden in organs of infected and immunized mice^39,49^. However, in our current study, we compared ChimeraT with the individual corresponding recombinant proteins and synthetic peptides, which was not done with either RCP1 or RCP2. Our new study data demonstrate clearly that ChimeraT was more efficient than the whole recombinant *Leishmania* source proteins.

On close examination of the humoral immune response in the current study, it was interesting to note that *L. infantum* infection of control mice (receiving saline or saponin alone) induced IgG antibodies that recognized SLA in ELISA. By contrast, parasite infection did not induce IgG antibodies that recognized ChimeraT protein. It is possible that parasite infection does not induce antibodies directed against the homologous protein T cell epitopes, and/or if any antibodies are induced, they have low affinity. Notably, we observed similarly low levels of anti-protein antibodies in control mice infected with *Leishmania* in our studies of the earlier chimera vaccines RCP1 and RCP2^39,49^.

Studies have used in silico immuno-bioinformatics tools to attempt the prediction of T-cell epitopes in *Leishmania* proteins^39,41^. Indeed, the design of vaccine candidates is being refined with increased knowledge of the nature of the antigens targeted by the mammalian immune response and by exploiting distinctive algorithms to predict and select molecules with the highest probability of inducing beneficial immune responses^56,57^. The selection of future vaccine candidates can also be done by studying immune responses on isolated PBMC from VL patients and healthy subjects^13,34^. In addition, thought needs to be given to an appropriate adjuvant to use with ChimeraT for human vaccination. Although saponin is a proven adjuvant for use in mice, it is no longer recommended for use in humans and therefore future studies with human-compatible adjuvants and delivery systems are certainly necessary. ChimeraT induced lymphoproliferation in PBMC cultures from VL patients after treatment and from healthy subjects, in addition to stimulating IFN-γ production. In this context, ChimeraT could be considered as a promising candidate for future translational studies in humans, due to the induction of markers of immunogenicity from cells isolated from non-exposed healthy individuals, as observed in previous studies with other candidate vaccine antigens^58–61^.

In conclusion, we have developed ChimeraT, a new polypeptide-based recombinant vaccine containing specific T-cell epitopes derived from four *Leishmania* antigenic and/or immunogenic proteins. Vaccination with ChimeraT/saponin induced a Th1-type immune response and reduced parasite loads in infected mice, and also induced the development of immune responses in PBMC from both VL patients after treatment and healthy subjects, characterized by a high lymphoproliferative response and IFN-γ production. In this context, ChimeraT deserves consideration as a promising candidate vaccine for human VL and potentially for VL in other mammalian hosts.

## Methods

### Construction of gene encoding ChimeraT protein

To construct the gene encoding ChimeraT, the amino acid sequences of the proteins Prohibitin (XP_001468827.1), eukaryotic initiation factor 5a (XP_001466105.1), *Leishmania* hypothetical protein 1 (LiHyp1, XP_001468941.1) and *Leishmania* hypothetical protein 2 (LiHyp2, XP_001462854.1) were searched using the SwissProt server (web.expasy.org/docs/swiss-prot). Prediction of the CD4^+^ and CD8^+^ T-cell epitopes were done with Rankpep (imed.med.ucm.es/Tools/rankpep.html)^62^ and confirmed by NetCTLPan 1.1 (http://www.cbs.dtu.dk/services/NetCTLpan/)^63^ and NetMHCII 2.3 (http://www.cbs.dtu.dk/services/NetMHCII/)^64^ servers, where the protein sequence was added in the input field, and the H-2Db, H-2Dd, H-2Kb, H-2Kd, H-2Kk and H-2Ld alleles from mouse and A2, A3, A24, and B7 alleles from human were evaluated to predict CD8^+^ T-cell epitopes; while the H-2IAb, H-2IAd, H-2Ias, H-2IEd and H-2IEb alleles from mouse and HLA-DR alleles from human were evaluated to predict the CD4^+^ T-cell epitopes. The selected alleles were those frequent in more than 90% of the human populations of any ethnic group, and in more than 90% of mice^39,65^. The Kolaskar & Tongaonkar Antigenicity method^66^, an online tool integrated into the immune epitope database analysis resource (IEDB) (http://tools.immuneepitope.org/tools/bcell/iedb_input), was used to predict epitope antigenicity, where a threshold value of 1.0 and window 25 were considered. The BepiPred 2.0 program was used to predict the B-cell epitopes, using a threshold value of 1.0^67^. Protein regions containing the combination of T-cell epitopes from humans and mice, but without B-cell epitopes, were selected to construct the chimeric protein. The following T-cell epitopes were selected: PHB (104-VRVLYQPNVENLYHIYRHIGVNAETVL-131), EIF5a (71-RLEDQAPSTHNVEVPFVKTFTYSVLDIQPNE-101), LiHyp1 (163-YIMSGPARYVYFHMVLPVEAQ-183) and LiHyp2 (416-NYDPNVWCAVPNCITCDRLDPSNR-439). To design the chimeric protein-encoding gene, the amino acids of each epitope were translated into the corresponding DNA coding sequence using the Standard Genetic Code Map and after conversion, the sequences were grouped linearly with two glycine residues as spacers between them to create a single protein. In the absence of a standard protocol to choose the relative positions of the epitopes within the chimera protein, the position of each epitope was chosen empirically to mimic their arrangement in their own source protein. This was done by analyzing where in the origin proteins the epitopes were located, i.e. whether they were closer to the C-terminus, N-terminus or within the center of the amino acid sequences. Therefore, the epitopes were ordered as follows: LiHyp1 epitope–GG–EIF5a epitope–GG–LiHyp2 epitope–GG–PHB epitope. Codon optimization for expression in *Escherichia coli* was done in silico with the web Codon Optimization tool (https://www.idtdna.com/CodonOpt) and optimized also to reduce the presence of intramolecular interactions of mRNA, which were calculated by using the MFOLD Program. The physicochemical characteristics of the ChimeraT and the individual T-cell epitopes were calculated with the ProtParam tool^68^. The ChimeraT-encoding gene and the individual peptides were synthesized commercially (GenScript®, USA).

### Cloning, expression, and purification of recombinant proteins

LiHyp1 (XP_001468941.1) and LiHyp2 (XP_001462854.1) genes and the ChimeraT protein were cloned commercially from *L. infantum* genomic DNA in the pET-28a(+)-TEV vector (Genscript®, USA), by using the primers: 5′-TCGAGGATCCATGCGCCAGCGAAAGCAC-3′ (forward) and 5′-GTCAAAGCTTTCACGCCTCCGGTAGCTG-3′ (reverse) for LiHyp1 and 5′-TCGAGGATCCATGAGCCCACACGGCGTC-3′ (forward) and 5′-GTCAGCGGCCGCTCAGTCCTCCTGACGTCG-3′ (reverse) for LiHyp2. To purify the rLiHyp1, rLiHyp2, and ChimeraT proteins, antigens were expressed in *E. coli* DE3 cells (Artic Express Agilent Technologies, USA) after induction with 0.1, 0.5 and 1.0 mM of isopropyl β-D-1-thiogalactopyranoside (IPTG), respectively, with shaking at 200 rpm for 24 h at 12 °C. The bacterial cells were ruptured by six cycles of sonication with 30 s each (38 MHz), followed by six cycles of freezing and thawing. Cellular debris was removed by centrifugation, and lysates for rLiHyp1, rLiHyp2 and ChimeraT proteins were passed through a HisTrap HP affinity column connected to an AKTA system (GE Healthcare Life Sciences, USA), and further purified on a Superdex^TM^ 200 gel-filtration column (GE Healthcare Life Sciences, USA).

*L. braziliensis* genomic DNA was used to amplify the nucleotide sequence encoding EIF5a (XP_001466105.1) using forward primer 5′-GCTAGCATGTCTGACGAGGACCAC-3′ and reverse primer 5′-AAGCTTCTACTTCTCCGCGGCATT-3′. The restriction sites for the NheI (GCTAGC) and HindIII (AAGCTT) restriction enzymes are underlined in the primer sequences. The PCR-amplified product was analyzed and excised from an agarose gel, purified, double-digested with NheI and HindIII, and ligated to a similarly digested pET28a-TEV vector. The recombinant plasmid was used to transform electrocompetent *E. coli* BL21 Arctic Express (DE3) cells (Agilent Technologies) by electroporation using a MicroPulser electroporation apparatus (Bio-Rad Laboratories). The recombinant protein was expressed by induction with 1.0 mM IPTG for 24 h at 12 °C with shaking at 200 rpm. Cells were lysed by sonication (applying continuous pulses of 30 s, with 15 s intervals, at 38 MHz) and centrifuged at 13,000 × *g* for 20 min at 4 °C. The recombinant protein was purified under non-denaturing conditions, using a 5 ml His-Trap column (GE Healthcare Life Science) attached to a fast protein liquid chromatography (FPLC) system (GE Healthcare Life Science)^37^.

The specific nucleotide sequence encoding for PHB protein (XP_001468827.1) was amplified by PCR from *L. infantum* kDNA by using the following primers: 5′-ACTAGTGGATCCATGGCGGCCGAGGCGCGGAAG-3′ (forward) and 5′-GGGCCCAAGCTTACTTCGCCCCGGAATGATC-3′ (reverse). The BamHI and NotI restriction enzymes were used. The DNA fragment was purified and ligated to pGEM®-T vector (Promega, USA) and the recombinant plasmid was used to transform *E. coli* XL1-Blue competent cells. The DNA fragment released after digestion of pGEM-rPHB was ligated into a pET28a-TEV vector, and used to transform *E. coli* BL21 cells (Agilent Technologies, USA). Expression of rPHB protein was induced with 0.5 mM IPTG for 3 h at 37 °C, at which time cells were ruptured by seven cycles of sonication (30 s each at 36 MHz), followed by six cycles of freeze and thaw. Cell debris was removed by centrifugation and rPHB was purified using a HisTrap HP affinity column connected to an AKTA system. The eluted fractions were concentrated in Amicon® ultra-15 centrifugal filters of 10,000 nominal molecular weight limit (NMWL, Millipore, Germany), and further purified on a Superdex^TM^ 200 gel-filtration column (GE Healthcare, USA)^34^.

After purification, all recombinant proteins were passed through a polymyxin-agarose column (Sigma-Aldrich, USA) to remove any residual endotoxin (<10 ng of lipopolysaccharide per 1 mg of protein, as quantified with the Quantitative Chromogenic Limulus Amebocyte Assay QCL-1000) (BioWhittaker, USA).

### Human blood samples

The study was approved by the Ethics Committee of the Federal University of Minas Gerais (UFMG; Belo Horizonte, Minas Gerais, Brazil), with protocol number CAAE–32343114.9.0000.5149. All participants provided written informed consent to take part in the study. Peripheral blood samples were collected from patients with visceral leishmaniasis (VL, *n* = 10; 6 males and 4 females, with ages ranging from 24 to 58 years), before and six months after treatment using pentavalent antimonials (Sanofi Aventis Farmacêutica Ltda, Suzano, São Paulo, Brazil). Infection was confirmed by PCR targeting *L. infantum* kDNA in their aspirates from spleen and/or bone marrow^69^. All patients received the same therapeutic schedule with antimonials (20 mg Sb^+5^ per kg during 30 days), and none presented any other infection or had any pre-existing clinical condition. Blood samples were also collected from healthy subjects (*n* = 10; 7 males and 3 females, with ages ranging from 24 to 45 years), and they did not present clinical signal of disease and exhibited negative serological results by the Kalazar Detect™ Test kit (InBios International®, USA). In addition, the healthy subjects were from the general population with no known previous history of contact with antigen aerosol.

### Parasites and preparation of soluble *Leishmania* antigen (SLA)

*L. infantum* (MHOM/BR/1970/BH46) strain was used. Stationary-phase promastigotes were grown at 24 °C in Schneider’s medium (Sigma-Aldrich, USA), supplemented with 20% (v/v) heat-inactivated fetal bovine serum (FBS; Sigma-Aldrich, USA), 20 mM L-glutamine, 100 U/mL penicillin and 50 µg/mL streptomycin pH 7.4.

To prepare SLA, 10^9^ stationary promastigotes were washed three times in cold sterile phosphate-buffered saline (PBS) pH 7.4. After six cycles of freezing and thawing, followed by ultrasonication (Ultrasonic processor, GEX600), with six cycles of 30 s at 38 MHz, the suspension was centrifuged at 9000 × *g* for 30 min at 4 °C, and the supernatant containing *L. infantum* SLA was collected. The protein concentration was estimated by the Bradford method^70^ and aliquots were stored at −80 °C until use.

### Lymphoproliferation assay and human cytokine production

The lymphoproliferative response was evaluated in peripheral blood mononuclear cell (PBMC) cultures from VL patients before and after treatment and healthy subjects^34^. PBMCs were purified from whole blood by Ficoll Hypaque density gradient centrifugation (GE Healthcare Bio-Sciences AB, Uppsala, Sweden). Cells (1 × 10^6^ per mL) were cultured in RPMI 1640 medium containing 20% (v/v) FBS, 2 mM L-glutamine, 200 U/mL penicillin, 100 µg/mL streptomycin, 50 μM 2-mercaptoethanol, 1 mM sodium pyruvate, and 1× non-essential amino acid, and then stimulated with synthetic peptide (20 µg/mL, each), recombinant protein (10 µg/mL, each) or *L. infantum* SLA (50 µg/mL), for 120 h at 37 °C in an atmosphere of 5%(v/v) CO_2_. Phytohemagglutinin (10 μg/mL; Sigma-Aldrich) was used as a positive stimulant control. At 102 h incubation, 20 µL of 5‐bromo‐2’‐deoxyuridine labeling solution was added, and the samples were incubated for 18 h at 37 °C, 5% (v/v) CO_2_. Lymphoproliferation was evaluated by ELISA, and the proliferation index was calculated by the ratio between the optical density values of the stimulated and unstimulated cultures.

IFN-γ and IL-10 levels were measured in the cell supernatants using commercial kits (BD OptEIA™ Human IFN-γ and IL-10 ELISA Set, BD Biosciences, USA), according to the manufacturer instructions. The detection sensitivity limit was 4.4 and 5.3 pg/mL for IFN‐γ and IL‐10 cytokines, respectively. Results were interpolated from a standard curve using reference recombinant cytokines.

### Immunization schedule and challenge infection

The study was approved by the Committee on the Ethical Handling of Research Animals of UFMG (code number 333/2015). The authors have complied with all relevant ethical regulations for animal testing and research. Female BALB/c mice (8 weeks old) were obtained from the breeding facilities of the Department of Biochemistry and Immunology, Institute of Biological Sciences, UFMG. Animals were vaccinated subcutaneously (*n* = 16 per group) in their left hind footpad (20 µL) with each recombinant protein (15 μg each) mixed with saponin (15 μg; *Quillaja saponaria* bark saponin, Sigma-Aldrich). Control animals received saline or saponin alone. Mice were immunized with three doses, each administered at 14 day intervals, and 30 days after the last immunization, animals (*n* = 8 per group) were euthanized and blood samples for serum preparation and spleens were collected for immunological evaluations. The remaining mice (*n* = 8 per group) were infected subcutaneously with 10^7^*L. infantum* stationary promastigotes in their right hind footpad.

### Evaluation of the parasite load

Forty-five days after infection, the remaining mice (*n* = 8 per group) were euthanized, and spleens, livers, bone marrow, and draining lymph nodes were removed for parasitological and immunological analyses. The parasite load was quantified by limiting-dilution technique. Briefly, organs were weighed and homogenized using a glass tissue grinder in sterile PBS. Tissue debris was removed by slow speed centrifugation (150 × *g*) and the cells were concentrated by centrifugation at 2000 × *g*. Next, the cells were suspended in 1 mL of supplemented Schneider’s medium, and 10-fold serial dilutions (from 10^−1^ to 10^−12^) were made in the same medium in 96-well flat-bottom microtiter plates (Nunc, Nunclon®, Denmark). Each individual sample was plated in triplicate and the presence of parasites was analyzed by microscopy after 7 days of culture at 24 °C. Pipette tips were discarded after each dilution step to avoid carrying adhered parasites from one well to another. Results were expressed as the log of the titer (i.e., the dilution corresponding to the last positive well) adjusted per milligram of organ^39^.

### Evaluating the cellular response

Spleen cells were collected 30 days after the last vaccine dose and 45 days after infection, and they were cultured as described previously^39^. Briefly, cells (5 × 10^6^ per mL) were maintained in DMEM (control) supplemented with 20% (v/v) FBS, 20 mM L-glutamine, 200 U/mL penicillin and 100 µg/mL streptomycin at pH 7.4, or separately stimulated with rPHB, rEIF5a, rLiHyp1, rLiHyp2 or ChimeraT (10 μg/mL each) used for immunization or with *L. infantum* SLA (50 μg/mL). In addition, the Chimera-T/saponin-group splenocytes were stimulated with the PHB, EIF5a, LiHyp1, and LiHyp2-derived epitopes (20 g/mL each). In all cases, plates were incubated for 48 h at 37 °C with 5% (v/v) CO_2_. Next, the cell supernatants were collected, and IFN-γ, IL-4, IL-10, IL-12, and GM-CSF cytokine levels were quantified by a capture ELISA (BD OptEIA TM set mouse kits, Pharmingen®, USA), following the manufacturer’s instructions.

Nitrite production was quantified in the supernatants from cultures of spleen cells isolated after infection with *L. infantum*. Spleen cells were stimulated with protein and SLA for 48 h (with medium alone as control) and culture supernatants (50 μL volumes) were mixed with an equal volume of Griess reagent (Sigma-Aldrich, USA) and incubated for 30 min at room temperature in the dark. Optical density (OD) values were measured in a microplate spectrophotometer (Molecular Devices, Spectra Max Plus, Canada) at 570 nm and nitrite concentrations were calculated from standard curve of known concentrations (100 µM to 0.78 mM) and data were expressed as μM Nitrite per 5 × 10^6^ cells^71^.

The involvement of CD4^+^ and CD8^+^ T-cell subtypes in the production of IFN-γ was evaluated in the ChimeraT/saponin group after infection. For this, spleen cells (5 × 10^6^ per mL) were in vitro stimulated with ChimeraT or *L. infantum* SLA (10 and 50 µg/mL, respectively) in the absence (medium, control) or presence of 5 μg/mL of anti-CD4 (GK 1.5), anti-CD8 (53-6.7) or anti-IL-12 (C17.8) monoclonal antibodies, for 48 h at 37 °C with 5% (v/v) CO_2_. The cell supernatant was then collected and IFN-γ levels were measured using a commercial kit. Appropriate isotype-matched controls—rat IgG2a (R35-95) and rat IgG2b (95-1)—were used. All antibodies (with no azide/low endotoxin^TM^) were purchased from BD (Pharmingen, USA).

### Assessment of antibody response

The protein and parasite-specific IgG1 and IgG2a isotype levels were evaluated in sera from parasite-challenged and/or vaccinated mice, which were collected 30 days after the last vaccine dose and 45 days after challenge. Briefly, titration curves were done to determine the most appropriate antigen concentration and antibody dilution to be used. Flexible microtiter immunoassay plates (Jetbiofil®, Brazil) were coated with the individual rPHB, rEIF5a, rLiHyp1, rLiHyp2 or ChimeraT proteins (0.5, 0.5, 1.0, 0.5, and 1.0 µg per well, respectively) or with *L. infantum* SLA (1.0 µg per well), all diluted in 100 µL/well of 50 mM carbonate coating buffer pH 9.6, for 18 h at 4 °C. Next, free binding sites were blocked using 250 µL of PBS plus 0.05% (v/v) Tween 20 and 5% (w/v) non-fat dry milk, for 1 h at 37 °C. After washing the wells five times with PBS-0.05% (v/v) Tween 20 (PBST), murine sera (100 µL/well of 1/100 diluted in PBST) were added to the wells and incubated for 1 h at 37 °C. Wells were washed as described above, and then incubated for 1 h at 37 °C with anti-mouse IgG1 and IgG2a horseradish-peroxidase conjugated antibodies (100 µL/well of 1/5,000 and 1/10,000 diluted in PBST, respectively) (Sigma-Aldrich, USA). After washing, reactions were developed by incubation for 30 min in the dark with a solution containing ortho-phenylenediamine and H_2_O_2_ (30 vol.) diluted in citrate-phosphate buffer, pH 5.0 (100 µL/well). Reactions were stopped by adding 2 N H_2_SO_4_, and optical density (OD) values were read in an ELISA microplate spectrophotometer (Molecular Devices, Spectra Max Plus, Canada), at λ492 nm^39^.

### Statistical analysis

Data were entered into Microsoft Excel (version 10.0) spreadsheets and analyzed using GraphPad Prism^TM^ (version 6.0 for Windows). Statistics were done using one-way analysis of variance (ANOVA), followed by Bonferroni´s post-test, which was used for multiple comparisons, and *P* values < 0.05 were considered significant. Data generated from the mouse immunization and infections and the evaluations of parasite load, as well as cellular and antibody responses, are representative of two independent experiments.

### Reporting summary

Further information on experimental design is available in the Nature Research Reporting Summary linked to this paper.

## Supplementary information

Reporting Summary

## Data Availability

All data generated or analyzed during this study are included in this published article.
