# Supplementary material for: A candidate vaccine for human visceral leishmaniasis based on a specific T cell epitope-containing chimeric protein protects mice against Leishmania infantum infection
Source: NPJ Vaccines. 2020 Aug 13;5:75. doi: 10.1038/s41541-020-00224-0 (PMC7426426; doi:10.1038/s41541-020-00224-0)
Supplement: Supplementary file 1 — Reporting Summary [file 41541_2020_224_MOESM1_ESM.pdf]

nature research

Corresponding author(s): NPJVACCINES-00608R

Last updated by author(s): 2020/09/06

Reporting Summary

Nature Research wishes to improve the reproducibility of the work that we publish. This form provides structure for consistency and transparency in reporting. For further information on Nature Research policies, see our Editorial Policies and the Editorial Policy Checklist.

Statistics

For all statistical analyses, confirm that the following items are present in the figure legend, table legend, main text, or Methods section.

n/a	Confirmed	
☐	☒	The exact sample size (n) for each experimental group/condition, given as a discrete number and unit of measurement
☐	☒	A statement on whether measurements were taken from distinct samples or whether the same sample was measured repeatedly
☐	☒	The statistical test(s) used AND whether they are one- or two-sided<br>*Only common tests should be described solely by name; describe more complex techniques in the Methods section.*
☐	☒	A description of all covariates tested
☒	☐	A description of any assumptions or corrections, such as tests of normality and adjustment for multiple comparisons
☐	☒	A full description of the statistical parameters including central tendency (e.g. means) or other basic estimates (e.g. regression coefficient) AND variation (e.g. standard deviation) or associated estimates of uncertainty (e.g. confidence intervals)
☒	☐	For null hypothesis testing, the test statistic (e.g. F, t, r) with confidence intervals, effect sizes, degrees of freedom and P value noted<br>*Give P values as exact values whenever suitable.*
☒	☐	For Bayesian analysis, information on the choice of priors and Markov chain Monte Carlo settings
☒	☐	For hierarchical and complex designs, identification of the appropriate level for tests and full reporting of outcomes
☒	☐	Estimates of effect sizes (e.g. Cohen's d, Pearson's r), indicating how they were calculated

Our web collection on statistics for biologists contains articles on many of the points above.

Software and code

Policy information about availability of computer code

Data collection	No software was used
Data analysis	Data were entered into Microsoft Excel (version 10.0) spreadsheets and analyzed using GraphPad PrismTM (version 6.0 for Windows).

For manuscripts utilizing custom algorithms or software that are central to the research but not yet described in published literature, software must be made available to editors and reviewers. We strongly encourage code deposition in a community repository (e.g. GitHub). See the Nature Research guidelines for submitting code & software for further information.

Data

Policy information about availability of data

All manuscripts must include a data availability statement. This statement should provide the following information, where applicable:

- Accession codes, unique identifiers, or web links for publicly available datasets
- A list of figures that have associated raw data
- A description of any restrictions on data availability

All data generated or analysed during this study are included in this published article.

Field-specific reporting

Please select the one below that is the best fit for your research. If you are not sure, read the appropriate sections before making your selection.

☒ Life sciences ☐ Behavioural & social sciences ☐ Ecological, evolutionary & environmental sciences

For a reference copy of the document with all sections, see nature.com/documents/nr-reporting-summary-flat.pdf

Life sciences study design

All studies must disclose on these points even when the disclosure is negative.

Sample size	Sample sizes for animal work based on previous published work to determine statistical significance per group size n=8.
Data exclusions	No exclusions
Replication	Allexperiments done n=3 times at least; this is stated throughout the Methods
Randomization	Not relevant to study
Blinding	Not relevant to study

Reporting for specific materials, systems and methods

We require information from authors about some types of materials, experimental systems and methods used in many studies. Here, indicate whether each material, system or method listed is relevant to your study. If you are not sure if a list item applies to your research, read the appropriate section before selecting a response.

Materials & experimental systems

n/a	Involved in the study	
☐	☒	Antibodies
☐	☒	Eukaryotic cell lines
☒	☐	Palaeontology and archaeology
☐	☒	Animals and other organisms
☐	☒	Human research participants
☒	☐	Clinical data
☒	☐	Dual use research of concern

Methods

n/a	Involved in the study	
☒	☐	ChIP-seq
☒	☐	Flow cytometry
☒	☐	MRI-based neuroimaging

Antibodies

Antibodies used	1. IFN-γ and IL-10 levels were measured in the cell supernatants using commercial kits (BD OptEIA™ Human IFN-γ and IL-10 ELISA Set, BD Biosciences, USA), according to the manufacturer instructions. These are antibody-based commercial ELISA.<br>2. IFN-gamma, IL-4, IL-10, IL-12 and GM-CSF cytokine levels were quantified by a capture ELISA (BD OptEIA TM set mouse kits, Pharmingen®, USA). Antibody-based commercial ELISA kits<br>3. anti-CD4 (GK 1.5), anti-CD8 (53-6.7) or anti-IL-12 (C17.8) monoclonal antibodies and appropriate isotype-matched controls – rat IgG2a (R35-95) and rat IgG2b (95-1) – were used. All antibodies (with no azide/low endotoxinTM) were purchased from BD (Pharmingen, USA).<br>4. anti-mouse IgG1 and IgG2a horseradish-peroxidase conjugated antibodies (Sigma-Aldrich, USA).
Validation	All kits are validated by their respective manufacturer; all details are in the Methods

Eukaryotic cell lines

Policy information about cell lines

Cell line source(s)	peripheral blood mononuclear cells isolated from human volunteers.
Authentication	none authenticated
Mycoplasma contamination	cell lines not tested (unnecessary)
Commonly misidentified lines (See ICLAC register)	None misidentified

Animals and other organisms

Policy information about studies involving animals; ARRIVE guidelines recommended for reporting animal research

Laboratory animals	Female BALB/c mice (8 weeks old) were obtained from the breeding facilities of the Department of Biochemistry and Immunology, Female BALB/c mice (8 weeks old) were obtained from the breeding facilities of the Department of Biochemistry and Immunology, Institute of Biological Sciences, UFMG, Brazil
Wild animals	did not involve wild animals
Field-collected samples	no samples collected from the field
Ethics oversight	The study was approved by the Committee on the Ethical Handling of Research Animals of UFMG (code number 333/2015).

Note that full information on the approval of the study protocol must also be provided in the manuscript.

Human research participants

Policy information about studies involving human research participants

Population characteristics	Peripheral blood samples were collected from patients with visceral leishmaniasis (VL, n=10; 6 males and 4 females, with ages ranging from 24 to 58 years), before and six months after treatment using pentavalent antimonials (Sanofi Aventis Farmacêutica Ltda, Suzano, São Paulo, Brazil). All patients received the same therapeutic schedule with antimonials (20 mg Sb+5 per kg during 30 days), and none presented any other infection or had any pre-existing clinical condition. Blood samples were also collected from healthy subjects (n=10; 7 males and 3 females, with ages ranging from 24 to 45 years), and they did not present clinical signal of disease and exhibited negative serological results by the Kalazar Detect™ Test kit (InBios International®, USA). In addition, the healthy subjects were from the general population with no known previous history of contact with antigen aerosol.
Recruitment	Recruited from general population (see characteristics above)
Ethics oversight	The study was approved by the Ethics Committee of the Federal University of Minas Gerais (UFMG; Belo Horizonte, Minas Gerais, Brazil), with protocol number CAAE–32343114.9.0000.5149.

Note that full information on the approval of the study protocol must also be provided in the manuscript.
